# “I Wish I Knew”: Assessing Older Adults’ Perceived and Actual Knowledge of Their Partners’ End-of-Life Preferences

**DOI:** 10.1093/geroni/igaf038

**Published:** 2025-04-24

**Authors:** Clément Meier, Maud Wieczorek, Carmen Borrat-Besson, Ralf J Jox, Jürgen Maurer

**Affiliations:** Faculty of Business and Economics (HEC), University of Lausanne, Lausanne, Switzerland; Swiss Centre of Expertise in the Social Sciences (FORS), Lausanne, Switzerland; Faculty of Business and Economics (HEC), University of Lausanne, Lausanne, Switzerland; Swiss Centre of Expertise in the Social Sciences (FORS), University of Lausanne, Lausanne, Switzerland; Palliative and Supportive Care Service, Chair in Geriatric Palliative Care, Lausanne University Hospital, Lausanne, Switzerland; Institute of Humanities in Medicine, Lausanne University Hospital and University of Lausanne, Lausanne, Switzerland; Faculty of Business and Economics (HEC), University of Lausanne, Lausanne, Switzerland

**Keywords:** Couples, Medical treatment, Partners’ wishes, Surrogates

## Abstract

**Background and Objectives:**

Despite the importance of surrogate decision-making by partners at the end of life, there is only limited research on older adults’ knowledge of their partners’ end-of-life preferences. Hence, this study investigates older adults’ perceived and actual knowledge of their partners’ preferences for end-of-life care and medical treatments.

**Research Design and Methods:**

We analyzed data from 667 respondents aged 50+ from Wave 8 (2019/2020) of the Survey on Health, Ageing, and Retirement in Europe in Switzerland. We assessed respondents’ actual knowledge by comparing their perceptions of their partners’ preferences for end-of-life care and medical treatments with the partners’ self-reported preferences. Additionally, respondents were asked to rate their perceived knowledge of their partners’ wishes. Associations were assessed using multivariable regression models, adjusting for social, health, and regional characteristics.

**Results:**

Respondents’ actual knowledge of their partners’ preferences varied, with the share of correct answers ranging from 35% to 81% depending on the preferences. More than 80% of respondents felt that they knew their partners’ end-of-life and medical treatment preferences “rather” or “very” well, and those respondents were more likely to identify their partners’ preferences accurately.

**Discussion and Implications:**

Improved communication between partners regarding their end-of-life preferences could significantly enhance surrogate end-of-life decision-making. However, since older adults’ perceived knowledge of their partners’ preferences seems overly optimistic, they may see little need to initiate such conversations by themselves, emphasizing the need for external educational interventions such as role-plays or case study discussions through, say, the community, or healthcare system to encourage such conversations.

**Translational Significance:** This study addresses the challenge of inaccurate surrogate decision-making among older adults regarding their partners’ end-of-life preferences. It reveals substantial gaps between perceived and actual knowledge of partners’ wishes, particularly for key aspects of end-of-life care. These findings highlight the importance of healthcare and community interventions to foster meaningful communication among couples. Approaches such as guided discussions and educational programs can enhance alignment between surrogate decisions and individuals’ true preferences, improving the quality of patient-centered care and alleviating emotional burdens for families.

Surrogate decision-making affects more than three-quarters of end-of-life situations and profoundly shapes end-of-life care and related patient experiences (Silveira et al., 2010; Torke et al., 2014). Medical ethics commonly stresses that end-of-life care should be both patient-centered and family-oriented (Beauchamp & Childress, 2019; Rosca et al., 2020). Surrogate decision-making, mostly by spouses or close family members (Su et al., 2020), is often seen as a valuable tool to reach these objectives in cases where patients have lost their decision-making capacity and advance directives or other forms of advance care planning outcomes are not readily available (Veloso & Tripodoro, 2016). While care practitioners commonly place significant trust in surrogates to determine the presumed preferences and values of patients without decision-making capacity, evidence suggests that surrogate decision-making is often not very accurate and may, thus, result in treatment and care arrangements that are inconsistent with patients’ own preferences and values (Moorman & Carr, 2008). Many of the key studies on surrogate decision-making date back more than a decade because large-scale empirical research on this specific issue has remained limited. These foundational studies continue to be widely cited due to their rigorous methodological approach and comprehensive assessments of surrogate accuracy, which remain highly relevant today.

The wishes of patients and the decisions made by surrogates often differ (Shalowitz et al., 2006). A review of studies conducted between 1966 and 2005 highlighted that surrogates, when placed in hypothetical decision-making scenarios, inaccurately predicted patients’ wishes approximately 32% of the time, frequently projecting their own preferences onto the patient (Ciroldi et al., 2007; Shalowitz et al., 2006). Surrogate decision-makers’ tendency to assume congruence between the surrogate’s and the patient’s preferences raises concerns about whether such surrogate decisions truly respect the patient’s own preferences and values. Recent evidence from a study during the COVID-19 pandemic finds that surrogates often inaccurately predict patient preferences and tend to choose more aggressive treatments than patients desire (R. L. Spalding & Edelstein, 2022). However, despite these inaccuracies, surrogates may still predict patient preferences more accurately than physicians, which suggests that close personal relationships may still be useful for obtaining valuable information for surrogate decision-making in cases where patients cannot make their own decisions anymore (Shalowitz et al., 2006).

The accuracy of surrogate decision-making does not appear to vary significantly across different patients and surrogate demographics or even by the status of the surrogate as a designated healthcare proxy (Moorman et al., 2009). Even prior discussions about end-of-life care preferences between the patient and the surrogate may not significantly increase the accuracy of surrogate decision-makers (Moorman et al., 2009). Discussions of patients and their surrogates regarding the patients’ end-of-life preferences only appear to result in a lower likelihood of surrogates reporting they do not know (Moorman & Carr, 2008). This finding is echoed in research involving a diverse cohort of couples, which revealed that individual engagement in advance care planning did not significantly predict the accuracy of reports on one’s partner’s healthcare preferences (Moorman & Inoue, 2013). These findings suggest that the effectiveness of surrogates in accurately revealing their partner’s healthcare preferences and values is not easily improved by formal or informal advance care planning activities (Meeker & Jezewski, 2005).

Challenges related to surrogate decision-making are also especially widespread and severe in intensive care units. While the majority of deaths in intensive care units result from decisions to withhold or withdraw life-sustaining treatments, only a very small fraction of patients are capable of participating in these decisions themselves, such that most decisions rely on some form of surrogate decision-making (Radwany et al., 2009). These decision-making situations often place an immense burden on surrogates, who must navigate the delicate balance between considerations of length of versus quality of life when deciding on patients’ treatment and care plans. Too often, surrogates seem to err on the side of very intensive and burdensome life-sustaining treatments in efforts to help extend a patient’s life (Ayalon et al., 2012; Batteux et al., 2020; Peterson et al., 2018; Wendler & Rid, 2011). What is more, surrogates experience greater emotional distress and decisional conflict when they are largely unaware of the patient’s own wishes and values regarding end-of-life treatments and care, which may increase their risk of regret or remorse after making these difficult decisions (Marx et al., 2014).

Given the persistent inaccuracies in surrogate decision-making, even when prior discussions have taken place, there is a critical need to better understand the factors influencing both perceived and actual knowledge of end-of-life preferences within couples (R. Spalding, 2021). This study addresses this gap by assessing both perceived and actual knowledge of partners’ wishes using data from a population-based sample in Switzerland. We compare individuals’ beliefs about their partners’ end-of-life and medical treatment preferences with their partners’ self-reported preferences, identifying areas of alignment and discordance. Additionally, we examine how perceived knowledge relates to actual accuracy, shedding light on potential overconfidence in surrogate decision-making. By highlighting key misalignments, this study provides important insights into the need for improved communication and targeted interventions to support more informed and patient-centered end-of-life decisions.

## Method

### Study Design and Participants

We use the Swiss data from Wave 8 of the Survey on Health, Ageing, and Retirement in Europe (SHARE) conducted between October 2019 and March 2020 (Börsch-Supan et al., 2013; SHARE-ERIC, 2024). SHARE is a longitudinal study gathering detailed information on the health, economic status, and social networks of individuals aged 50 and above, along with their partners, in 27 European countries and Israel. For Wave 8, 2,005 individuals in Switzerland and their partners were interviewed in person, with the majority of these, that is, 1,891 respondents (94.3%), also completing a supplementary Switzerland-specific paper-and-pencil questionnaire on end-of-life issues, which was administered at the end of the face-to-face interview. For the purposes of this study, we only selected participants whose partners also participated in SHARE Wave 8—the only survey wave that includes questions on perceived and actual knowledge of partners’ wishes—resulting in a final sample of 950 participants (475 couples). We further restricted the sample to those for whom we had complete data on the outcome and exposure variables, as well as other covariates used in the analysis. This sample selection resulted in a final sample size of 667 respondents.

During the preparation of this work, the author(s) used OpenAI’s ChatGPT and Grammarly in order to assist with the grammatical refinement of the paper. After using these tools/services, the authors reviewed and edited the content as needed and take full responsibility for the content of the publication.

### Outcome Variable

#### End-of-life and medical treatment preferences

**Figure 1.**
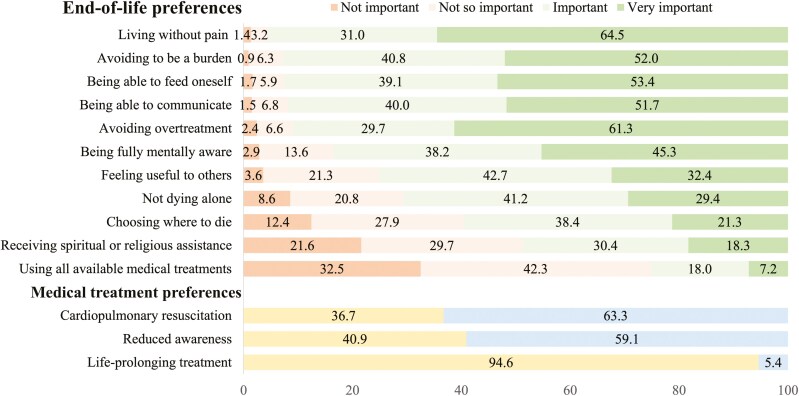
Distribution of end-of-life and medical treatment preferences, adults aged 50+, SHARE Switzerland, 2019/2020, *n* = 667.

The supplementary Switzerland-specific questionnaire on end-of-life issues included a list of 14 end-of-life preferences for the last six months of life, derived from the scientific literature on individuals’ perspectives of what constitutes a “good death” (Borrat-Besson, Vilpert, Borasio, & Maurer, 2022; Meier et al., 2016; Steinhauser et al., 2000). Respondents were asked to evaluate the end-of-life preferences domains in terms of their importance on a four-point scale: “very important,” “important,” “not so important,” and “not important.” The end-of-life preferences ranged from the importance of feeling useful and avoiding being a burden, to decisions about the location of death, not dying alone, discussing fears, receiving spiritual support, avoiding overtreatment, maintaining physical contact and communication, self-feeding, utilizing all medical treatments to prolong life, living without pain, and remaining mentally aware during the final six months of life. The complete list of end-of-life preferences is available in Figure 1 and Supplementary Material Section 1. Additionally, respondents were asked to express their preferences for three medical treatments for which preferences are often explicitly elicited in advance directives forms in Switzerland (Vilpert et al., 2023). The first question asked whether respondents would wish to be resuscitated in the event of a cardiac and/or respiratory arrest in their current health state; the second inquired whether they would prefer to forgo all life-prolonging measures if they were incapacitated following an accident, stroke, or heart attack, with physicians considering it unlikely that they would regain capacity; and the third asked whether they would be willing to accept to enter a state of reduced awareness in the event of a disease-causing unbearable pain and symptoms such as fear, restlessness, breathing difficulties, and nausea (see Supplementary Material Section 2).

#### Actual knowledge of partners’ end-of-life and medical treatment preferences

In the same questionnaire on end-of-life issues, respondents who had a spouse or partner were asked to consider their partner’s perspective on 11 of the previously mentioned 14 end-of-life preferences. These included feeling useful, avoiding being a burden, choosing the place of death, not dying alone, receiving spiritual support, avoiding overtreatment, maintaining communication, self-feeding, utilizing life-prolonging treatments, living pain-free, and remaining mentally aware (Supplementary Material Section 3). Respondents were asked to report their partner’s preferences using the same four-point scale: “*very important*,” “*important*,” “*not so important*,” and “*not important*.” Additionally, they were tasked with assessing their partner’s preferences for the three previously mentioned medical treatments in scenarios of cardiac or respiratory arrest, incapacitation without recovery, and severe pain and symptoms (Supplementary Material Section 4). Dichotomized variables were created to determine the accuracy of respondents’ assessments of their partners’ preferences, with a 1 indicating a correct assessment and a 0 indicating an incorrect assessment. For the end-of-life preferences, another set of dichotomized variables was used to assess the accuracy of the respondents’ assessments. Then, another set of dichotomized variables was generated with a score of 1 if the respondent’s assessment aligned with their partner’s general tendency of the preference, categorizing responses as either “*very important/important*” or “*not so important/not important*.” For example, a respondent would score 1 if both they and their partner rated a preference as either “*very important*” or “*important*,” even if the stated categories do not match exactly, such as when the respondent indicates that he/she thinks that a given end-of-life domain is “*very important*” for his/her partner while the partner indicates that this domain is only “*important*” for her/him. Similarly, a score of 1 was given if both the respondent and their partner considered a preference domain as either “*not so important*” or “*not important*”, even if the statements did not match exactly. A score of 0 was assigned otherwise. Finally, three accuracy scores were developed: *the general accuracy score*, which awarded one point for each correct assessment of an end-of-life preference, with a potential range from 0 to 11; *the treatment accuracy score*, which also awarded one point for each correct assessment of a medical treatment preference, with a potential range from 0 to 3; and *the accuracy score tendency*, which awarded one point for correctly assessing the general tendency of each end-of-life preference, with a potential range from 0 to 11. The accuracy score tendency accounts for cases where respondents did not exactly match their partners” responses but correctly identified the general direction of their preferences. Responses were grouped into two broad categories: (a) “*important*” (combining “*very important*” and “*important*”) and (b) “*not important*” (combining “*not so important*” and “*not important*”). If the respondent placed their partner’s preference in the same category as the partner’s self-reported response, they received one point, even if their exact answer differed (e.g., selecting “very important” when the partner selected “important”).

### Exposure

#### Perceived knowledge of partners’ end-of-life and medical treatment preferences

The national paper-and-pencil questionnaire administered in Wave 8 included a set of questions where respondents were asked to rate their knowledge of their partners’ end-of-life wishes (Supplementary Material Section 5). Specifically, respondents were asked two questions regarding how well they believe to know (a) their partner’s general end-of-life wishes and (b) their partner’s preferences for medical treatment at the end of life. Both questions allowed for four potential responses: “very well,” “rather well,” “not very well,” and “not at all.” Due to the relatively low frequency of the categories “not very well” and “not at all,” we combined these two categories for a total of three response options overall: “very well,” “rather well,” and “not very well/not at all.”

### Covariates

The statistical analysis adjusted for various demographic and socioeconomic factors. These encompassed sex (identified as either male or female), age (divided into three categories: 50–64 years, 65–74 years, and 75 years and above), and levels of education (defined as low for International Standard Classification of Education [ISCED] levels 0-1-2; medium for ISCED levels 3–4; and high for ISCED levels 5–6; Hoffmeyer-Zlotnik & Wolf, 2003). In addition, linguistic regions within Switzerland (German-speaking, French-speaking, or Italian-speaking), subjective financial situation (assessed by the level of ease in making end of the month meet: easily, fairly easily, or with difficulty), type of residence area (urban vs. rural), and self-reported health condition (rated as either poor/fair, good, or very good/excellent) were also included in the analysis.

### Statistical Analysis

The characteristics of the study population were described using numerical counts and relative frequencies. A first stacked bar chart was used to show the distribution of respondents’ preferences for end-of-life and medical treatment. Another stacked bar chart was used to show the distribution of respondents’ actual knowledge of their partners’ preferences. Specifically, this chart shows the relative frequency of (a) correct exact answers, (b) correct tendency, and (c) incorrect answers. In the case of medical treatment preferences, respondents also had a “don’t know” option as a potential response category. A final stack bar chart was used to present the distribution of partners’ perceived knowledge regarding end-of-life and medical treatment preferences. Partial associations between respondents’ perceived knowledge of the partners’ end-of-life and medical treatment preferences and the three accuracy scores were assessed using separate ordinary least squares regression models. Each multivariable regression model adjusted for sex, age, education levels, Switzerland’s linguistic regions, subjective financial difficulties, type of residence area, and self-rated health. Furthermore, the error terms were clustered by household to accommodate for possible unseen interdependencies between the primary respondents and their partners. Statistical analyses were conducted using STATA/SE 18.0 software (STATA Corporation, College Station, TX). Two-sided *p* values < .05 were considered statistically significant.

## Results

Table 1 presents the demographic and socioeconomic profile of the study participants, consisting of adults over the age of 50 years from the SHARE Switzerland dataset of 2019/2020, with a total sample size of 667. The final analytical sample consists of 49.6% males and 50.4% females. In terms of age, 34.8% of participants are between 50 and 64 years, 42.2% are in the 65–74-year age range, and 23.0% of our respondents are 75 and older. Regarding educational attainment, 14.1% of the respondents have a low education level, the majority, at 60.7%, have a medium level of education, and 25.2% possess a high education level. Concerning respondents’ subjective financial status, 58.6% reported they could make ends meet easily, and 30.1% fairly easily, while 11.3% reported some difficulties in making ends meet. The majority of the sample speaks German (71.8%), followed by French speakers (26.2%), and a small minority speaking Italian (2.0%). In terms of residency, 61.6% live in rural areas as opposed to 38.4% in urban areas. Concerning self-assessed health, 15.0% rated their health as poor or fair, 41.1% as good, and a plurality of 43.9% as very good or excellent. The accuracy scores, based on a scale of end-of-life preferences, show an average general accuracy score of 5.1 (out of a maximum of 11), with a standard deviation of 2. The treatment accuracy score averages at 1.9 (out of a maximum of 3) with a standard deviation of 0.9. The accuracy score tendency averages at 8.6 (out of a maximum of 11), with a standard deviation of 1.7.

**Table 1.** Characteristics of the Study Population, Adults Aged 50+, SHARE Switzerland, 2019/2020, *n* = 667

Variables	*n*	%	Mean (*SD*)
Gender			
Male	331	49.6	
Female	336	50.4	
Age groups			
50–64 years	232	34.8	
65–74 years	282	42.2	
75+ years	153	23.0	
Education			
Low	94	14.1	
Middle	405	60.7	
High	168	25.2	
Make ends meet			
Easily	391	58.6	
Fairly easily	201	30.1	
With difficulty	75	11.3	
Language			
German	479	71.8	
French	175	26.2	
Italian	13	2.0	
Living area			
Urban	256	38.4	
Rural	411	61.6	
Self-rated health			
Poor/fair health	100	15.0	
Good health	274	41.1	
Very good/excellent health	293	43.9	
General accuracy score (range: 0–11)			5.1 (2.0)
Treatment accuracy score (range: 0–3)			1.9 (0.9)
Accuracy score tendency (range: 2–11)			8.6 (1.7)

*Notes*: Number of observations for the whole sample. *SD* = standard deviation.

Figure 1 shows the distribution of end-of-life preferences, the majority of individuals consider living “without pain” (64.5%) and “avoiding being a burden” (52.0%) as very important. “Being able to feed oneself” and “being able to communicate” are also highly valued, with more than 90% of the respondents marking these as important or very important. The desire to avoid overtreatment is notably significant, with 61.3% of individuals considering it very important. Similarly, “being fully mentally aware” is a priority for many, receiving a high importance rating from 45.3% of respondents. However, “feeling useful to others” and “not dying alone” are perceived with lesser urgency, though still important for a substantial proportion of participants. “Choosing where to die” and “receiving spiritual or religious assistance” are considered less critical, with only 21.3% and 18.3% of individuals, respectively, deeming them very important. Regarding the medical treatment preferences, there is a resounding preference to forgo life-prolonging treatments when there is no hope of recovery, with a striking 94.6% of participants against it. Conversely, “using all available medical treatments” is less favored, with only 7.2% considering it very important. Attitudes toward “reduced awareness” also indicate a preference for less aggressive treatments, with a significant number leaning toward accepting reduced awareness (59.1%). Finally, there is a clear majority inclination toward “cardiopulmonary resuscitation (CPR),” with 63.3% of participants expressing a preference for CPR. Overall, the graphical representation underscores the prevailing preferences that prioritize comfort, autonomy, and less invasive treatments.

**Figure 2.**
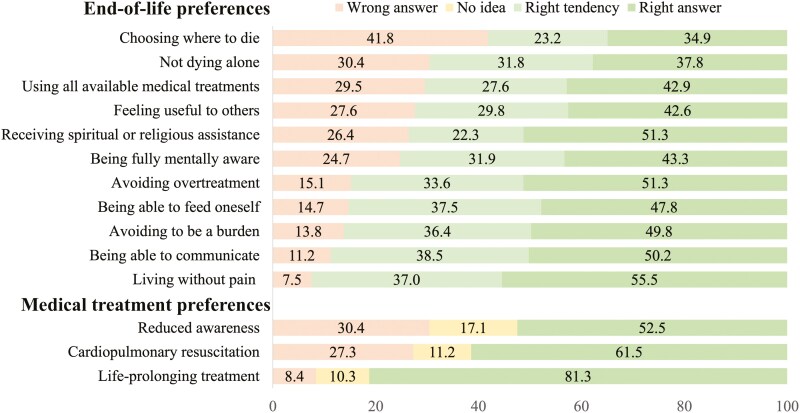
Distribution of actual knowledge of partners’ end-of-life and medical treatment preferences, adults aged 50+, SHARE Switzerland, 2019/2020, *n* = 667.

To assess respondents’ accuracy in identifying their partners’ end-of-life and medical treatment preferences, we classified their responses into four categories: (a) Right answer—the respondent’s answer exactly matched their partner’s self-reported preference; (b) Right tendency—the respondent’s answer did not match exactly but correctly identified the general direction of their partner’s preference (e.g., selecting “very important” when the partner selected “important”); (c) Wrong answer—the respondent’s answer was neither exact nor aligned with the general tendency of their partner’s preference; and (d) No idea—the respondent explicitly selected “I have no idea” (an option available for medical treatment preferences only). Figure 2 illustrates the distribution of respondents’ actual knowledge of their partners’ end-of-life and medical treatment preferences. The dimensions in Figure 2 are presented in order of the share of “incorrect answers,” which refer to respondents’ reports on their partners preferences that even fail to capture the right tendency of the partners’ true underlying preferences and which, therefore, create scope for surrogate end-of-life care that may be very poorly aligned with the dying person’s actual end-of-life preferences. For our broader measures of end-of-life preferences, the least accurately assessed preference was “choosing where to die,” with 41.8% of respondents providing the wrong answer. This was followed by “not dying alone” with 30.4% incorrect answers, and “using all available medical treatments” at 29.5%. Additionally, 27.6% of respondents incorrectly assessed the preference for “feeling useful to others,” while 26.4% were wrong about “receiving spiritual or religious assistance.” Preferences such as “being fully mentally aware” and “avoiding overtreatment” had incorrect assessment rates of 24.7% and 15.1%, respectively. The ability “to feed oneself” and “avoiding being a burden” were wrongly assessed by 14.7% and 13.8% of respondents, respectively. The least amount of incorrect answers was observed for “being able to communicate” and “living without pain,” with 11.2% and 7.5% incorrect responses, respectively. In terms of medical treatment preferences, 30.4% of respondents incorrectly identified their partners’ preferences for “reduced awareness,” and 27.3% were wrong about “cardiopulmonary resuscitation.” The preference for “life-prolonging treatment” had the lowest incorrect assessment rate, with only 8.4% of respondents providing the wrong answer. Figure 2 also presents the percentage of respondents who had the “right tendency” in their answers, those who had “no idea” about their partners’ preferences, and those who provided the right answer.

Additional details regarding the direction of potential misalignment between perceived and actual knowledge of partners’ end-of-life and medical treatment preferences are available in Supplementary Material Section 6. This section provides a detailed comparison of the tabulated results, illustrating the discrepancies and congruencies between what respondents believe their partners prefer and the partners’ actual preferences. For instance, the section shows that there is a significant discrepancy in the perceptions of choosing where to die, with 66.2% of individuals who perceive it as very important incorrectly assessing their partners’ views as either important, not so important, or not important. This discrepancy increases as the perceived importance of choosing where to die decreases, reaching 90.4% incorrect assessments for those who perceive it as not important.

**Figure 3.**
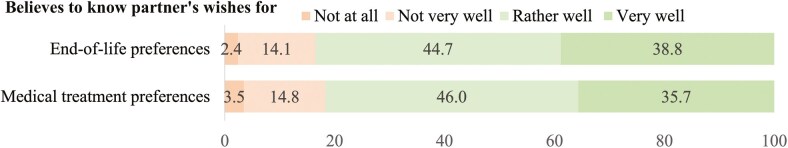
Distribution of perceived knowledge of partners’ end-of-life and medical treatment preferences, adults aged 50+, SHARE Switzerland, 2019/2020, *n* = 667.

Figure 3 displays the distribution of the subjective assessments of respondents on how well they believe to know their partner’s end-of-life and medical treatment preferences. Regarding end-of-life preferences, 38.8% of respondents report knowing their partner’s wishes “very well,” while 44.7% felt they knew them “rather well.” Regarding medical treatment preferences, 35.7% of respondents stated to know their partner’s wishes “very well,” with 46.0% believing to know them “rather well.” In both categories, only a minority of respondents admitted to “not very well” knowing their partner’s preferences, with 14.1% for end-of-life preferences and 14.8% for medical treatment preferences, while only very few respondents states to “not at all” know their partner’s preferences (2.4% for end-of-life preferences and 3.5% for medical treatment preferences). Overall, most people seem confident regarding their own knowledge of their partners’ end-of-life and medical treatment preferences.

In Table 2, partial associations between respondents’ perceived knowledge of their partners’ preferences, respondents’ accuracy scores, and various covariates are presented. Respondents who reported knowing their partner’s wishes for end-of-life care “very well” had higher accuracy scores compared to those who reported knowing their partners’ preferences “not very well/not at all” [Average Marginal Effect (AME): 0.66, *p* < .01]. A similar pattern was observed for medical treatment preferences, where knowing “rather well” and “very well” partners’ medical treatment preferences showed positive associations with higher accuracy scores (AME: 0.43, *p* < .001 for “rather well”; AME: 0.52, *p* < .001 for “very well”). Notably, the estimations showed no significant differences in the accuracy of partner assessments across different demographics and socioeconomic factors.

**Table 2.** Partial Associations Between Actual Knowledge of Partners’ Preferences on Perceived Knowledge and Covariates, Adults Aged 50+, SHARE Switzerland, 2019/2020, *n* = 667

Variables	General accuracy score	Treatment accuracy score	Accuracy score tendency
Gender (male)			
Female	−0.03	0.00	−0.24^*^
	(0.14)	(0.06)	(0.11)
Age group (50–64 years)			
65–74 years	0.10	−0.01	−0.05
	(0.19)	(0.08)	(0.16)
75+ years	0.28	−0.02	0.06
	(0.27)	(0.10)	(0.21)
Education (low)			
Secondary	0.25	0.02	0.03
	(0.27)	(0.10)	(0.21)
Tertiary	−0.06	0.18	−0.14
	(0.31)	(0.12)	(0.25)
Language (German)			
French	−0.06	−0.07	0.21
	(0.19)	(0.09)	(0.16)
Italian	0.32	−0.25	0.12
	(0.71)	(0.28)	(0.66)
Living area (urban)			
Rural	−0.06	−0.03	0.02
	(0.18)	(0.07)	(0.14)
Self-rated health (bad health)			
Good health	−0.10	0.09	0.35
	(0.24)	(0.10)	(0.20)
Very good/excellent health	−0.10	0.10	0.38
	(0.26)	(0.11)	(0.20)
Believes to know partner’s wishes for the end-of-life in general (not very well/not at all)			
Rather well	0.18		0.03
	(0.23)		(0.20)
Very well	0.66^**^		0.36
	(0.25)		(0.21)
Believes to know partner’s wishes for medical treatment at the end-of-life (not very well/not at all)			
Rather well		0.43^***^	
		(0.10)	
Very well		0.52^***^	
		(0.11)	
Observations	667	667	667

*Notes*: The table shows average marginal effects and standard errors in parentheses. The columns show the results from ordinary least squares regressions of accuracy scores of partner’s preferences on subjective awareness and the covariates. The covariates include sex, age, education levels, subjective financial situation, linguistic region, living area, and self-rated health.

^*^ *p* < .05. **p < .01. ***p < .001.

## Discussion

Using data from 667 adults aged 50 and older in Switzerland, this study investigates the extent to which older adults perceive and know their partners’ end-of-life and medical treatment preferences. A strength of this research is its inclusion of many different end-of-life and medical treatment preferences that capture a wide range of important domains concerning end-of-life experiences and care, thereby providing a robust framework for understanding and evaluating surrogate decision-making in this area. Our findings first reveal widespread errors in individuals’ perceptions of their partners’ end-of-life preferences, especially in areas such as “choosing where to die,” “not dying alone,” and “using all available medical treatments.” At the same time, our data further show that most respondents seem to think that they know their partners’ end-of-life and medical preferences reasonably well, which may lead to a low perceived need for related communication within the couple. While individuals who reported having good knowledge of their partners’ end-of-life preferences were generally more accurate in assessing their partners’ preferences, important misconceptions still remained among these respondents. Those who believed they knew their partners’ preferences had incorrect answers ranging from 7.3% (importance of living without pain) to 42% (choosing where to die), depending on the specific preference. These findings underscore the critical role of end-of-life communication between partners, even in couples where the partners may not perceive a need for such conversations due to their high perceived knowledge of their partners’ preferences. Additionally, the inconsistencies observed in some patient preferences—for example, rejecting life-prolonging treatments while still opting for CPR—may contribute to surrogate confusion, making it even more difficult to accurately infer a partner’s true wishes. This underscores the need for clearer communication and structured discussions that help individuals articulate consistent and well-informed preferences.

### Novel Insights Into Older Adults’ Knowledge of Their Partners’ End-of-Life Preferences

While a few studies investigate surrogate decision-making by partners at the end of life, research on older adults’ perceived and actual awareness of their partners’ end-of-life preferences is still rather limited. Firstly, existing studies often only consider a rather limited number of care characteristics or treatments when considering concordance between individuals’ end-of-life preferences and values and their partners’ perceptions of these preferences and values (Moorman et al., 2009; Shalowitz et al., 2006). By contrast, our study explores the accuracy of individuals’ perceptions of their partner’s end-of-life preferences much more broadly using data on 14 major dimensions of end-of-life care and treatment dimensions covering not just medical but also psychosocial and other important dimensions of end-of-life care (Borrat-Besson et al., 2022; Borrat-Besson, Vilpert, Borasio, & Maurer, 2022). Moreover, our study also allows us to assess the perceived awareness of older adults regarding their partners’ end-of-life preferences as well as the relationship of this perceived awareness with actual accuracy in predicting partners’ stated end-of-life preferences. Our study, therefore, provides important novel insights into older adults’ knowledge regarding their partners’ end-of-life care and treatment preferences for a wide range of end-of-life dimensions as well as their perceived knowledge of these preferences along with information on whether higher perceived knowledge of the partner’s end-of-life preferences is actually positively correlated with higher preference prediction accuracy or not.

### Actual Knowledge of Partners’ Preferences

The results showed significant variability in respondents’ actual knowledge of their partners’ preferences for end-of-life care and medical treatment. Overall, accuracy was higher for medical treatment preferences compared to more general end-of-life aspects, though part of this difference may be related to slight differences in the question formats pertaining to these two different domains. In previous work by our research group, four key dimensions underlying end-of-life preferences were identified: a medical dimension (such as pain management and maintaining abilities), a psychosocial dimension (such as social and spiritual support), a control dimension, and a burden dimension (Borrat-Besson et al., 2022). Based on these four dimensions, it seems that respondents tend to be more accurate in assessing some of these dimensions than others. For instance, assessment accuracy was highest in the medical dimension, particularly for “life-prolonging treatment,” with 81.3% of respondents accurately assessing their partners’ preferences. This finding suggests that respondents are relatively well-informed or in agreement about decisions that have a direct impact on life duration. In the psychosocial dimension, which includes aspects of social and spiritual support, 51.3% of respondents accurately assessed their partners’ preference for “receiving spiritual or religious assistance.” However, the control dimension, which relates to preferences around autonomy and decision-making at the end of life, had lower accuracy in the assessment of partners’ preferences, as seen with “choosing where to die,” indicating less certainty or discussion in these areas. The burden dimension, reflecting wishes like “not to be a burden” and “to feel useful,” had moderate accuracy in partners’ preference assessment. This could suggest that while respondents are aware of their partner’s end-of-life preferences to avoid being a burden, they may not have detailed discussions about the specifics of this aspect, such as emotional burdens. In summary, the variability in accuracy of assessing partners’ preferences across different end-of-life dimensions highlights the nuanced understanding partners have of each other’s end-of-life preferences, with generally higher assessment accuracy in medical aspects and greater variability in the control and burden dimensions. Finally, the results showed no significant variations across different demographics and socioeconomic factors, which stands in contrast to findings from two other studies. Zettel-Watson et al. (2008) found that wives generally outperformed husbands in predicting their spouses’ healthcare wishes, attributing this to gender-related differences in healthcare experiences and roles within the family. Similarly, Batteux et al. (2019) observed that older participants were more likely to know their partners’ preferences.

### Perceived Knowledge of Partners’ Preferences

Our results reveal a positive correlation between how well individuals believe they know their partner’s wishes regarding end-of-life and medical treatment preferences and their actual preferences, which suggests that perceived knowledge of the partners’ end-of-life and medical treatment preferences have some signaling value for objective accuracy. At the same time, even among partners with high perceived knowledge, significant inaccuracies and thus a need for related communication persists. A previous study exploring the quality of the relationship between partners found that surrogates reporting higher levels of family conflict showed lower accuracy in predicting their partners’ preferences (Parks et al., 2011). These findings highlight that fostering a strong, communicative relationship may be critical as it could increase confidence in one’s understanding of a partner’s preferences and, thus, a more accurate assessment. Better alignment between perceived and actual wishes may ultimately ensure that end-of-life care honors the true desires and values of the individuals involved.

### Practical Implications and Future Research

Despite the importance of advance care planning, physicians and caregivers often lack awareness of patients’ end-of-life wishes, emphasizing the urgent need for improved communication strategies to ensure that patient preferences are respected (Calvache et al., 2021). Healthcare providers should facilitate earlier and more frequent conversations between partners about end-of-life care preferences, which should not only include medical aspects of end-of-life care but also broader psychosocial aspects related to this challenging life stage. One strategy is to integrate these discussions as a routine part of health maintenance visits for older adults. Providers could offer “conversation starters” materials and guide couples through potential scenarios, encouraging them to share values and wishes with each other. Creating a supportive environment within healthcare settings that normalizes these discussions can be beneficial (Groebe et al., 2019). This could be done by ensuring that staff are trained to recognize opportunities for initiating conversations about advance care planning and by providing resources such as brochures, worksheets, and digital tools that couples can use to continue these conversations at home. Previous studies have shown that even though prior discussions about end-of-life care preferences or engagement in advance care planning are not directly associated with the accuracy of predicting partners’ end-of-life preferences (Meeker & Jezewski, 2005; Moorman & Inoue, 2013; Moorman et al., 2009), engagement in various planning activities enables surrogates to make more precise substituted judgments (Batteux et al., 2019; Inoue & Moorman, 2015). In addition, a study investigating whether a physician-led discussion about advance directives improves the accuracy of surrogate decision-makers found that it may enhance surrogate preparedness in understanding patients’ end-of-life care preferences (Martins et al., 2022). Moreover, educational interventions in the community could include workshops, online modules, and community seminars that focus on the importance of advance care planning. These interventions should aim to improve understanding of the medical, legal, and emotional aspects of end-of-life decisions. To help couples become more aware of each other’s end-of-life preferences, structured educational programs can provide valuable support. For instance, the End of Life Aid Skills for Everyone (EASE) course, offered by the Scottish Partnership for Palliative Care, equips participants with the knowledge and confidence to navigate conversations about death, dying, and bereavement through group discussions, short films, and peer support activities *End of Life Aid Skills for Everyone (EASE). (2024)*. Similarly, the “Last Aid” course, an international initiative originating in Germany and now available in multiple countries, provides essential training on palliative care, emotional support, and end-of-life communication, helping individuals feel more prepared to discuss and respect their loved ones’ wishes *NO LABEL (2024)*. These programs highlight the role of public education in fostering open dialogue and ensuring that end-of-life preferences are better understood and honored. In addition, programs that use role-playing or case studies to simulate decision-making scenarios could help individuals and couples better understand the complexities of surrogate decision-making. One effective icebreaker to initiate conversations between partners about end-of-life preferences is the DöBra cards, a tool developed in Sweden to facilitate discussions on end-of-life values and care preferences. These cards provide a structured yet flexible approach that encourages deep reflection beyond just medical decisions, making it easier for partners to express their wishes and concerns (Johansson, 2022). Additionally, education on how to document and communicate preferences formally, for instance, through living wills or advanced directives, should be part of such interventions. In future research, these strategies and interventions could be studied longitudinally to determine their effectiveness in improving the accuracy of surrogate decision-making, thereby informing continuous improvement in this critical area of healthcare and education.

### Limitations

Our study acknowledges a number of limitations. Firstly, the complexity and personal nature of end-of-life preferences mean that there could be nuances or important end-of-life aspects not fully captured by the survey questions used in the study. Additionally, while SHARE tries for an accurate representation of Switzerland’s older adult population, factors such as attrition in longitudinal research and nonresponses to certain items may affect our findings, potentially overestimating the percentage of individuals who accurately assess their partner’s end-of-life preferences. However, the strong response rate in the Swiss questionnaire and the uniform traits of those excluded provide a degree of reliability to our conclusions. Moreover, the potential issue of ‘cooperation’ among partners when completing the survey together could lead to overstated accuracy in respondents’ knowledge of their partners’ preferences. Finally, another limitation is the cross-sectional nature of the study, which captures preferences at a single point in time and cannot track changes in older adults’ perceived and actual knowledge of their partners’ end-of-life preferences over time. Future research could aim to address these limitations by incorporating longitudinal designs, qualitative data, and more diverse population samples to provide a more comprehensive understanding of the factors influencing surrogate decision-making accuracy.

## Conclusion

Our study highlights significant knowledge gaps among older adults in assessing their partner’s preferences for the end of life. These gaps seem especially large for psychosocial aspects of end-of-life care but are also relevant with regard to medical aspects. Moreover, while older adults’ actual knowledge of their partner’s end-of-life preferences is associated with how well they believe they know those preferences, significant knowledge gaps even persist in the group of adults that state good knowledge of their partners’ preferences and who may, therefore, only have low perceived needs to engage more frequently or deeper into conversations about this topic with their partner. Therefore, fostering conversations about end-of-life options could be a key factor in improving the precision of surrogate decision-making. This underscores the potential for healthcare practitioners or other stakeholders such as community organizations and advocacy groups to develop targeted communication strategies that prompt such discussions, which may contribute to better end-of-life care outcomes. Educational interventions designed to improve dialogue between partners about end-of-life preferences also emerge as a vital tool for enhancing surrogate decision-making accuracy. Future research is necessary in the context of aging populations and the increasing importance of surrogate decision-making in healthcare. This research should particularly focus on longitudinal studies that can track changes in older adults’ perceived and actual knowledge of their partners’ end-of-life preferences over time, and qualitative research that can explore the nuances of communication and relationship dynamics. Ultimately, this study provides a foundation upon which both practical interventions and future research can build, with the goal of ensuring that end-of-life care honors the true desires and values of those it serves.

## Supplementary Material

igaf038_suppl_Supplementary_Materials_1

## Data Availability

This paper uses data from SHARE-ERIC (2024). Survey of Health, Ageing and Retirement in Europe (SHARE) Wave 8. Release version: 9.0.0. SHARE-ERIC. Data set. DOI: 10.6103/SHARE.w8.900. Study data already de-identified are available to the scientific community upon submitting a data requisition application to the SHARE study.
