# Supplementary material for: “I Wish I Knew”: Assessing Older Adults’ Perceived and Actual Knowledge of Their Partners’ End-of-Life Preferences
Source: Innov Aging. 2025 Apr 24;9(6):igaf038. doi: 10.1093/geroni/igaf038 (PMC12199151; doi:10.1093/geroni/igaf038)
Supplement: igaf038_suppl_Supplementary_Materials_1 [file igaf038_suppl_supplementary_materials_1.docx]

***Innovation in Aging* Supplementary Material: Meier et al. “I wish I knew”: Assessing older adults’ perceived and actual knowledge of their partners’ end-of-life preferences.**

| **Section 1: End-of-life preferences** |
| --- |
| People differ in their opinion about what is important to ensure they are spending the last months of their lives as best as possible. How important are each of the following potential end-of-life aspects for you when  thinking about the last six months of life?  Answer categories: "Very important", "Important", "Not so important", "Not important"   1. Feeling useful to others (giving time, sharing knowledge, etc.) 2. Avoiding being a burden on family 3. Planning the events following my death (funeral announcement…) 4. Choosing where I die 5. Not dying alone 6. Talking about my fears 7. Receiving spiritual or religious assistance 8. Avoiding overtreatment 9. Having physical contact (e.g. holding hands) 10. Being able to talk or communicate with others 11. Being able to feed myself 12. Using all available medical treatments to prolong life until the end 13. Living without pain 14. Being fully mentally aware |

| **Section 2: Medical treatment preferences** |
| --- |
| **Question 1:** Imagine that you experience a cardiac and/or respiratory arrest. In this situation, you wish…  Answer categories:   1. "to be resuscitated" 2. "not to be resuscitated"   **Question 2:** Imagine that you are incapacitated following an accident, a stroke, or a heart attack. After initial emergency measures and careful medical assessment, physicians deem it very unlikely that you will regain capacity. In this situation, you preferer…  Answer categories:   1. "to forgo all measures which would only serve to prolong your life and suffering" 2. "that, despite the poor outlook, every medically appropriate measure should be taken"   **Question 3:** Imagine that you suffer from a disease that causes unbearable pain and symptoms such as fear restlessness, breathing difficulties and nausea. In this situation, …  Answer categories:   1. "you wish to receive optimum treatment of pain and other distressing symptoms and you are prepared to accept the reduced awareness (sedation) which such treatment may induce." 2. "for you, alertness and the ability to communicate are more important than optimal relief of pain and other symptoms." |

| **Section 3: Respondents’ knowledge of partners’ end-of-life preferences** |
| --- |
| We have listed here again end-of-life aspects that may be important to ensure they are spending the last months of their lives as best as possible. We would like you now to report for each of the listed aspects what your partner or spouse would consider as very important, important, not so important or not important when she/he thinks about the last six months of her/his life. If you don’t know, please give us your best estimate.  According to me, my partner or my spouse would consider that …  Answer categories: "Very important", "Important", "Not so important", "Not important"   1. Feeling useful to others (giving time, sharing knowledge, etc.) 2. Avoiding being a burden on family 3. Choosing where I die 4. Not dying alone 5. Receiving spiritual or religious assistance 6. Avoiding overtreatment 7. Being able to talk or communicate with others 8. Being able to feed myself 9. Using all available medical treatments to prolong life until the end 10. Living without pain 11. Being fully mentally aware |

| **Section 4: Respondents’ knowledge of partners’ medical treatment preferences** |
| --- |
| **Question 1:** Imagine that your partner or spouse experiences a cardiac and/or respiratory arrest. In this situation, according to you, he/she would wish…  Answer categories:   1. "to be resuscitated" 2. "not to be resuscitated" 3. “I have no idea”   **Question 2:** Imagine that you partner or spouse is incapacitated following an accident, a stroke, or a heart attack. After initial emergency measures and careful medical assessment, physicians deem it very unlikely that he/she will regain capacity. In this situation, according to you, he/she would preferer…  Answer categories:   1. "to forgo all measures which would only serve to prolong his/her life and suffering" 2. "that, despite the poor outlook, every medically appropriate measure should be taken" 3. “I have no idea”   **Question 3:** Imagine that your partner or your spouse suffers from a disease that causes unbearable pain and symptoms such as fear restlessness, breathing difficulties and nausea. In this situation, according to you, …  Answer categories:   1. "he/she would wish to receive optimum treatment of pain and other distressing symptoms and be prepared to accept the reduced awareness (sedation) which such treatment may induce." 2. "for him/her, alertness and the ability to communicate are more important than optimal relief of pain and other symptoms." 3. “I have no idea” |

| **Section 5: Perceived knowledge of partners’ end-of-life and medical treatment preferences** |
| --- |
| **Question 1:** How well do you think that you know your spouse’s or your partner’s wishes…  Answer categories: "Very well", "Rather well", "Not very well", "Not at all"   1. for the end of life in general? 2. For medical treatment at the end-of-life? |

| **Section 6: Tabulation of actual versus perceived end-of-life and medical treatment preferences among partners in a household** |
| --- |
| First row has *frequencies* and second row has *row percentages*  **Choosing where to die**   \| Partner's actually stated importance of choosing where to die \| Perceived partners’ importance of choosing where to die \| \| \| \| \| \| --- \| --- \| --- \| --- \| --- \| --- \| \|  \| Very important \| Important \| Not so important \| Not important \| Total \| \| Very important \| 48 \| 53 \| 27 \| 14 \| 142 \| \|  \| 33.80 \| 37.32 \| 19.01 \| 9.86 \| 100.00 \| \| Important \| 50 \| 112 \| 73 \| 21 \| 256 \| \|  \| 19.53 \| 43.75 \| 28.52 \| 8.20 \| 100.00 \| \| Not so important \| 36 \| 70 \| 60 \| 20 \| 186 \| \|  \| 19.35 \| 37.63 \| 32.26 \| 10.75 \| 100.00 \| \| Not important \| 8 \| 30 \| 32 \| 13 \| 83 \| \|  \| 9.64 \| 36.14 \| 38.55 \| 15.66 \| 100.00 \| \| Total \| 142 \| 265 \| 192 \| 68 \| 667 \| \|  \| 21.29 \| 39.73 \| 28.79 \| 10.19 \| 100.00 \| \|  \| \| \| \| \| \|   **Not dying alone**   \| Partner's actually stated importance of not dying alone \| Perceived partners’ importance of not dying alone \| \| \| \| \| \| --- \| --- \| --- \| --- \| --- \| --- \| \|  \| Very important \| Important \| Not so important \| Not important \| Total \| \| Very important \| 96 \| 81 \| 16 \| 3 \| 196 \| \|  \| 48.98 \| 41.33 \| 8.16 \| 1.53 \| 100.00 \| \| Important \| 110 \| 125 \| 35 \| 5 \| 275 \| \|  \| 40.00 \| 45.45 \| 12.73 \| 1.82 \| 100.00 \| \| Not so important \| 42 \| 59 \| 28 \| 10 \| 139 \| \|  \| 30.22 \| 42.45 \| 20.14 \| 7.19 \| 100.00 \| \| Not important \| 15 \| 28 \| 11 \| 3 \| 57 \| \|  \| 26.32 \| 49.12 \| 19.30 \| 5.26 \| 100.00 \| \| Total \| 263 \| 293 \| 90 \| 21 \| 667 \| \|  \| 39.43 \| 43.93 \| 13.49 \| 3.15 \| 100.00 \| \|  \| \| \| \| \| \|   **Using all available medical treatments**   \| Partner's actually stated importance of using all available medical treatments \| Perceived partners’ importance of using all available medical treatments \| \| \| \| \| \| --- \| --- \| --- \| --- \| --- \| --- \| \|  \| Very important \| Important \| Not so important \| Not important \| Total \| \| Very important \| 12 \| 13 \| 17 \| 6 \| 48 \| \|  \| 25.00 \| 27.08 \| 35.42 \| 12.50 \| 100.00 \| \| Important \| 10 \| 49 \| 44 \| 17 \| 120 \| \|  \| 8.33 \| 40.83 \| 36.67 \| 14.17 \| 100.00 \| \| Not so important \| 12 \| 61 \| 149 \| 60 \| 282 \| \|  \| 4.26 \| 21.63 \| 52.84 \| 21.28 \| 100.00 \| \| Not important \| 14 \| 26 \| 101 \| 76 \| 217 \| \|  \| 6.45 \| 11.98 \| 46.54 \| 35.02 \| 100.00 \| \| Total \| 48 \| 149 \| 311 \| 159 \| 667 \| \|  \| 7.20 \| 22.34 \| 46.63 \| 23.84 \| 100.00 \| \|  \| \| \| \| \| \|   **Feeling useful to others**   \| Partner's actually stated importance of feeling useful to others \| Perceived partners’ importance of feeling useful \| \| \| \| \| \| --- \| --- \| --- \| --- \| --- \| --- \| \|  \| Very important \| Important \| Not so important \| Not important \| Total \| \| Very important \| 97 \| 96 \| 20 \| 3 \| 216 \| \|  \| 44.91 \| 44.44 \| 9.26 \| 1.39 \| 100.00 \| \| Important \| 91 \| 149 \| 38 \| 7 \| 285 \| \|  \| 31.93 \| 52.28 \| 13.33 \| 2.46 \| 100.00 \| \| Not so important \| 32 \| 67 \| 35 \| 8 \| 142 \| \|  \| 22.54 \| 47.18 \| 24.65 \| 5.63 \| 100.00 \| \| Not important \| 4 \| 13 \| 4 \| 3 \| 24 \| \|  \| 16.67 \| 54.17 \| 16.67 \| 12.50 \| 100.00 \| \| Total \| 224 \| 325 \| 97 \| 21 \| 667 \| \|  \| 33.58 \| 48.73 \| 14.54 \| 3.15 \| 100.00 \| \|  \| \| \| \| \| \|   **Receiving spiritual or religious assistance**   \| Partner's actually stated importance of receiving spiritual or religious assistance \| Perceived partners’ importance of receiving spiritual or religious assistance \| \| \| \| \| \| --- \| --- \| --- \| --- \| --- \| --- \| \|  \| Very important \| Important \| Not so important \| Not important \| Total \| \| Very important \| 70 \| 29 \| 20 \| 3 \| 122 \| \|  \| 57.38 \| 23.77 \| 16.39 \| 2.46 \| 100.00 \| \| Important \| 37 \| 88 \| 61 \| 17 \| 203 \| \|  \| 18.23 \| 43.35 \| 30.05 \| 8.37 \| 100.00 \| \| Not so important \| 11 \| 46 \| 101 \| 40 \| 198 \| \|  \| 5.56 \| 23.23 \| 51.01 \| 20.20 \| 100.00 \| \| Not important \| 5 \| 13 \| 43 \| 83 \| 144 \| \|  \| 3.47 \| 9.03 \| 29.86 \| 57.64 \| 100.00 \| \| Total \| 123 \| 176 \| 225 \| 143 \| 667 \| \|  \| 18.44 \| 26.39 \| 33.73 \| 21.44 \| 100.00 \|   **Being fully mentally aware**   \| Partner's actually stated importance of being fully mentally aware \| Perceived partners’ importance of being fully mentally aware \| \| \| \| \| \| --- \| --- \| --- \| --- \| --- \| --- \| \|  \| Very important \| Important \| Not so important \| Not important \| Total \| \| Very important \| 143 \| 124 \| 28 \| 7 \| 302 \| \|  \| 47.35 \| 41.06 \| 9.27 \| 2.32 \| 100.00 \| \| Important \| 84 \| 129 \| 39 \| 3 \| 255 \| \|  \| 32.94 \| 50.59 \| 15.29 \| 1.18 \| 100.00 \| \| Not so important \| 29 \| 44 \| 17 \| 1 \| 91 \| \|  \| 31.87 \| 48.35 \| 18.68 \| 1.10 \| 100.00 \| \| Not important \| 7 \| 8 \| 4 \| 0 \| 19 \| \|  \| 36.84 \| 42.11 \| 21.05 \| 0.00 \| 100.00 \| \| Total \| 263 \| 305 \| 88 \| 11 \| 667 \| \|  \| 39.43 \| 45.73 \| 13.19 \| 1.65 \| 100.00 \| \|  \| \| \| \| \| \|   **Avoiding overtreatment**   \| Partner's actually stated importance of avoiding overtreatment \| Perceived partners’ importance of avoiding overtreatment \| \| \| \| \| \| --- \| --- \| --- \| --- \| --- \| --- \| \|  \| Very important \| Important \| Not so important \| Not important \| Total \| \| Very important \| 238 \| 133 \| 25 \| 13 \| 409 \| \|  \| 58.19 \| 32.52 \| 6.11 \| 3.18 \| 100.00 \| \| Important \| 87 \| 90 \| 14 \| 7 \| 198 \| \|  \| 43.94 \| 45.45 \| 7.07 \| 3.54 \| 100.00 \| \| Not so important \| 11 \| 18 \| 13 \| 2 \| 44 \| \|  \| 25.00 \| 40.91 \| 29.55 \| 4.55 \| 100.00 \| \| Not important \| 9 \| 4 \| 2 \| 1 \| 16 \| \|  \| 56.25 \| 25.00 \| 12.50 \| 6.25 \| 100.00 \| \| Total \| 345 \| 245 \| 54 \| 23 \| 667 \| \|  \| 51.72 \| 36.73 \| 8.10 \| 3.45 \| 100.00 \| \|  \| \| \| \| \| \|   **Being able to feed oneself**   \| Partner's actually stated importance of being able to feed oneself \| Perceived partners’ importance of being able to feed oneself \| \| \| \| \| \| --- \| --- \| --- \| --- \| --- \| --- \| \|  \| Very important \| Important \| Not so important \| Not important \| Total \| \| Very important \| 171 \| 155 \| 26 \| 4 \| 356 \| \|  \| 48.03 \| 43.54 \| 7.30 \| 1.12 \| 100.00 \| \| Important \| 94 \| 141 \| 25 \| 1 \| 261 \| \|  \| 36.02 \| 54.02 \| 9.58 \| 0.38 \| 100.00 \| \| Not so important \| 9 \| 23 \| 7 \| 0 \| 39 \| \|  \| 23.08 \| 58.97 \| 17.95 \| 0.00 \| 100.00 \| \| Not important \| 9 \| 1 \| 1 \| 0 \| 11 \| \|  \| 81.82 \| 9.09 \| 9.09 \| 0.00 \| 100.00 \| \| Total \| 283 \| 320 \| 59 \| 5 \| 667 \| \|  \| 42.43 \| 47.98 \| 8.85 \| 0.75 \| 100.00 \| \|  \| \| \| \| \| \|   **Avoiding to be a burden**   \| Partner's actually stated importance of avoiding to be a burden \| Perceived partners’ importance of avoiding to be a burden \| \| \| \| \| \| --- \| --- \| --- \| --- \| --- \| --- \| \|  \| Very important \| Important \| Not so important \| Not important \| Total \| \| Very important \| 178 \| 152 \| 14 \| 3 \| 347 \| \|  \| 51.30 \| 43.80 \| 4.03 \| 0.86 \| 100.00 \| \| Important \| 90 \| 146 \| 35 \| 1 \| 272 \| \|  \| 33.09 \| 53.68 \| 12.87 \| 0.37 \| 100.00 \| \| Not so important \| 7 \| 27 \| 8 \| 0 \| 42 \| \|  \| 16.67 \| 64.29 \| 19.05 \| 0.00 \| 100.00 \| \| Not important \| 2 \| 3 \| 1 \| 0 \| 6 \| \|  \| 33.33 \| 50.00 \| 16.67 \| 0.00 \| 100.00 \| \| Total \| 277 \| 328 \| 58 \| 4 \| 667 \| \|  \| 41.53 \| 49.18 \| 8.70 \| 0.60 \| 100.00 \|   **Being able to communicate**   \| Partner's actually stated importance of being able to communicate \| Perceived partners’ importance of being able to communicate \| \| \| \| \| \| --- \| --- \| --- \| --- \| --- \| --- \| \|  \| Very important \| Important \| Not so important \| Not important \| Total \| \| Very important \| 191 \| 143 \| 9 \| 2 \| 345 \| \|  \| 55.36 \| 41.45 \| 2.61 \| 0.58 \| 100.00 \| \| Important \| 111 \| 138 \| 17 \| 1 \| 267 \| \|  \| 41.57 \| 51.69 \| 6.37 \| 0.37 \| 100.00 \| \| Not so important \| 17 \| 22 \| 6 \| 0 \| 45 \| \|  \| 37.78 \| 48.89 \| 13.33 \| 0.00 \| 100.00 \| \| Not important \| 3 \| 4 \| 3 \| 0 \| 10 \| \|  \| 30.00 \| 40.00 \| 30.00 \| 0.00 \| 100.00 \| \| Total \| 322 \| 307 \| 35 \| 3 \| 667 \| \|  \| 48.28 \| 46.03 \| 5.25 \| 0.45 \| 100.00 \| \|  \| \| \| \| \| \|   **Living without pain**   \| Partner's actually stated importance of living without pain \| Perceived partners’ importance of living without pain \| \| \| \| \| \| --- \| --- \| --- \| --- \| --- \| --- \| \|  \| Very important \| Important \| Not so important \| Not important \| Total \| \| Very important \| 264 \| 154 \| 11 \| 1 \| 430 \| \|  \| 61.40 \| 35.81 \| 2.56 \| 0.23 \| 100.00 \| \| Important \| 92 \| 102 \| 13 \| 0 \| 207 \| \|  \| 44.44 \| 49.28 \| 6.28 \| 0.00 \| 100.00 \| \| Not so important \| 8 \| 9 \| 4 \| 0 \| 21 \| \|  \| 38.10 \| 42.86 \| 19.05 \| 0.00 \| 100.00 \| \| Not important \| 5 \| 3 \| 1 \| 0 \| 9 \| \|  \| 55.56 \| 33.33 \| 11.11 \| 0.00 \| 100.00 \| \| Total \| 369 \| 268 \| 29 \| 1 \| 667 \| \|  \| 55.32 \| 40.18 \| 4.35 \| 0.15 \| 100.00 \| \|  \| \| \| \| \| \|   **Cardiopulmonary resuscitation**   \| Partner's wishes in case of cardiac and/or respiratory arrest \| Believed partner's wishes in case of cardiac and/or respiratory arrest \| \| \| \| \| --- \| --- \| --- \| --- \| --- \| \|  \| To be resuscitated \| Not to be resuscitated \| No idea \| Total \| \| To be resuscitated \| 309 \| 66 \| 47 \| 422 \| \|  \| 73.22 \| 15.64 \| 11.14 \| 100.00 \| \| Not to be resuscitated \| 116 \| 101 \| 28 \| 245 \| \|  \| 47.35 \| 41.22 \| 11.43 \| 100.00 \| \| Total \| 425 \| 167 \| 75 \| 667 \| \|  \| 63.72 \| 25.04 \| 11.24 \| 100.00 \| \|  \| \| \| \| \|   **Life-prolonging treatment**   \| Partner's wishes in case of incapacity without hope to recover \| Believed partner's wishes in case of incapacity without hope to recover \| \| \| \| \| --- \| --- \| --- \| --- \| --- \| \|  \| To forgo measures that prolong life \| To take every appropriate measures \| No idea \| Total \| \| To forgo measures that prolong life \| 532 \| 36 \| 63 \| 631 \| \|  \| 84.31 \| 5.71 \| 9.98 \| 100.00 \| \| To take every appropriate measures \| 20 \| 10 \| 6 \| 36 \| \|  \| 55.56 \| 27.78 \| 16.67 \| 100.00 \| \| Total \| 552 \| 46 \| 69 \| 667 \| \|  \| 82.76 \| 6.90 \| 10.34 \| 100.00 \|   **Reduced awareness**   \| Partner's wishes in case of unbearable pain and symptoms \| Believed partner's wishes in case of unbearable pain and symptoms \| \| \| \| \| --- \| --- \| --- \| --- \| --- \| \|  \| Accept reduced awareness \| Refuse reduced awareness \| No idea \| Total \| \| Accept reduced awareness \| 243 \| 88 \| 63 \| 394 \| \|  \| 61.68 \| 22.34 \| 15.99 \| 100.00 \| \| Refuse reduced awareness \| 115 \| 107 \| 51 \| 273 \| \|  \| 42.12 \| 39.19 \| 18.68 \| 100.00 \| \| Total \| 358 \| 195 \| 114 \| 667 \| \|  \| 53.67 \| 29.24 \| 17.09 \| 100.00 \| \|  \|  \|  \|  \|  \| |
